# Efficacy of Once Daily Darunavir/Ritonavir in PI-Naïve, NNRTI-Experienced Patients in the ODIN Trial

**DOI:** 10.1155/2015/962574

**Published:** 2015-08-18

**Authors:** Anna Maria Geretti, Mathe Moeketsi, Ralph Demasi, Yvon van Delft, Perry Mohammed

**Affiliations:** ^1^University of Liverpool, Liverpool, UK; ^2^Folateng Ward, Sebokeng Hospital, Gauteng, South Africa; ^3^Janssen Research and Development, LLC, Titusville, NJ, USA; ^4^Janssen Global Public Health, Tilburg, Netherlands; ^5^Janssen Global Public Health, High Wycombe, UK

## Abstract

*Background*. An exploratory subanalysis of the ODIN trial was performed to evaluate the efficacy of darunavir/ritonavir (DRV/r) 800/100 mg OD versus 600/100 mg BID in patients who were NNRTI-experienced but PI-naïve. *Methods*. ODIN was a phase III, 48-week study comparing DRV/r OD versus BID in 590 treatment-experienced patients with no DRV resistance-associated mutations (RAMs) at screening. Patients received DRV/r 800/100 mg OD or DRV/r 600/100 mg BID plus ≥2 NRTIs. Of the 590 patients randomized, 272 (46%) were NNRTI-experienced but PI-naïve. *Results*. Overall, 272 patients received DRV/r OD (*n* = 135) or BID (*n* = 137) plus ≥2 optimised NRTIs. The mean age was 39 years; 35% were female; 27% were Black, 24% Caucasian, 26% Oriental/Asian, and 23% other races; 17% were recruited in South Africa; and 48% had non-B HIV-1 subtypes. Mean baseline plasma HIV-1 RNA load was 4.10 log_10_⁡ copies/mL; median CD4 cell count was 258 cells/*μ*L. At week 48, 111/135 (82%) of DRV/r OD and 109/137 (80%) of DRV/r BID patients achieved an HIV-1 RNA load <50 copies/mL. No patient developed primary PI RAMs. *Conclusion*. DRV/r 800/100 mg OD in combination with ≥2 optimised NRTIs led to virological suppression <50 copies/mL in 82% of NNRTI-experienced, PI-naïve patients by week 48.

## 1. Introduction

Approximately 12 million people with HIV infection in developing countries are taking two nucleoside reverse transcriptase inhibitors (NRTIs) and a nonnucleoside reverse transcriptase inhibitor (NNRTI) for first-line treatment (e.g., tenofovir/lamivudine/efavirenz) [1]. If NNRTI-based regimens fail, there is a high risk of drug resistance to both classes of antiretroviral agents [2, 3]. In developed countries, resistance testing is used to guide selection of second-line options, but this is not practical for widespread use in resource-limited settings.

The standard second-line antiretroviral treatment (ART) recommended by the World Health Organization (WHO) for resource-limited settings is two NRTIs and a ritonavir-boosted protease inhibitor (PI/r) [4]. The two PI/r currently used in developing countries are lopinavir/ritonavir (LPV/r) and atazanavir/ritonavir (ATV/r) [4]. Both protease inhibitors are available for relatively low costs, in heat stable formulations [5]. In treatment-naïve patients, DRV/r 800/100 mg once daily has shown greater virological efficacy than LPV/r, with a lower risk of lipid elevations and gastrointestinal side effects [6, 7]. In treatment-experienced patients, DRV/r 600/100 mg BID has also shown improved virological efficacy and safety compared with LPV/r, with a reduced risk of treatment-emergent drug resistance [8]. However in the 2LADY study, conducted in West Africa, the efficacy of LPV/r and DRV/r was similar in protease inhibitor naïve, treatment-experienced patients. In this study, the 800/100 mg once daily dose of DRV/r was used [9].

A large randomized trial comparing ATV/r with DRV/r in treatment-naïve patients has shown virological benefits of DRV/r 800/100 mg once daily versus atazanavir/ritonavir [10]. ATV/r has not been studied extensively in treatment-experienced patients; one trial comparing ATV/r versus LPV/r showed slightly lower rates of plasma HIV-1 RNA suppression in the ATV/r arm, but the trial was underpowered to show noninferiority [11]. Taken together, these observations may raise concern that the activity of ATV/r-based second-line treatment regimens can be weakened by extensive preexisting NRTI resistance, but controlled data are needed to substantiate the hypothesis.

The ODIN trial evaluated the efficacy and safety of DRV/r in treatment-experienced patients who had no DRV resistance-associated protease mutations at baseline. The trial compared two DRV/r doses: 800/100 mg once daily and 600/100 mg twice daily in combination with at least two NRTIs and showed overall noninferiority of the OD arm compared with the BID arm [12, 13].

If DRV/r is to be proposed as part of second-line ART regimens in low-income countries, it will be mainly used in PI-naïve patients who have experienced failure of first-line NNRTI-based therapy. Since the ODIN trial included both PI-naïve and PI-experienced subjects, the objective of this exploratory analysis was to evaluate the efficacy and safety of DRV/r in the subset of patients who were NNRTI-experienced but PI-naïve at study entry and relate the findings to the activity of the NRTI backbone, thereby providing evidence to guide treatment choices for second-line therapy.

## 2. Methods

The methods of the ODIN trial have been described in detail elsewhere [12]. Briefly, the trial recruited 590 patients who were receiving stable combination ART for at least 12 weeks, were experiencing virological failure, and had no DRV RAMs. Patients with active AIDS defining illnesses, pregnant or breastfeeding women, or people with Grade 3 or Grade 4 laboratory abnormalities were not allowed to enter the study. Patients were randomized to DRV/r at doses of either 800/100 mg OD or 600/100 mg BID. Patients also received at least two optimized NRTIs, based on the results of genotypic resistance testing at screening. Use of other antiretrovirals was not allowed during the study. The primary objective of the trial was to demonstrate noninferior efficacy for the OD treatment arm, compared to the BID arm. Plasma HIV-1 RNA measurements and safety assessments were performed at screening, at baseline, and at each study visit (weeks 4, 8, 12, 24, 36, and 48 and withdrawal). During the trial, genotypic resistance testing was performed on samples with confirmed detectable (≥50 copies/mL) HIV-1 RNA copies/mL.

Resistance mutations were scored according to Virtual Phenotype (Tibotec, Beerse, Belgium). The activity of the NRTIs used for each patient was calculated by assigning a value of 1 for drugs with full susceptibility and 0 for those with any resistance.

All patients signed written informed consent and the protocol was approved by local and national ethics committees.

In this analysis, we describe the efficacy and safety of the patients who had received no protease inhibitors before baseline. The percentage of patients with HIV-1 RNA suppression below 50 or 400 copies/mL was analysed using the Time to Loss of Virological Response (TLOVR) algorithm. Clinical and laboratory adverse events were graded by severity using the Division of AIDS (DAIDS) grading scale. The analyses were exploratory in nature. The trial had not been powered to compare the treatment groups for this subset of patients.

## 3. Results

### 3.1. Baseline Characteristics

Of the 272 PI-naïve patients in the ODIN trial, 135 received DRV/r 800/100 mg OD and 137 received DRV/r 600/100 mg BID. The baseline characteristics were well-balanced between the arms (Table 1). The median age was 38 years (range of 18–77) and 35% of patients were female. The baseline CD4 count was below 200 cells/*μ*L in 42% of patients and the baseline HIV-1 RNA level was at least 100,000 copies/mL in 8%. Overall, 27% of the patients were Black, 26% were Oriental or Asian, and 42% were recruited in either Africa or Asian countries. A range of HIV-1 subtypes were found in the patients. The most common HIV-1 subtype was B (52%), but 19% of patients had HIV-1 subtype C and a further 26% of patients had recombinant subtypes (CRF01_AE, CRF02_AG, or CRF012_BF). The most common NRTIs used in DRV/r 800/100 mg OD and the DRV/r 600/100 mg BID arms comprised tenofovir (85.9% and 82.5% resp.), zidovudine (72.6% and 66.4%), and lamivudine (51.1% and 60.6%), whereas use of stavudine (18.5% and 16.8%), emtricitabine (14.8% and 16.1%), abacavir (14.1% and 12.4%), and didanosine (3.7% and 5.1%) was less common. For patients with genotypic results available at baseline, the percentage with 0, 1, or ≥2 sensitive NRTIs in their randomised treatment was well-balanced between the treatment arms (Table 4). There were 14 patients who did not have a genotype available at their baseline visit and who were not included in this analysis.

### 3.2. Virological Responses

**Figure 1:**
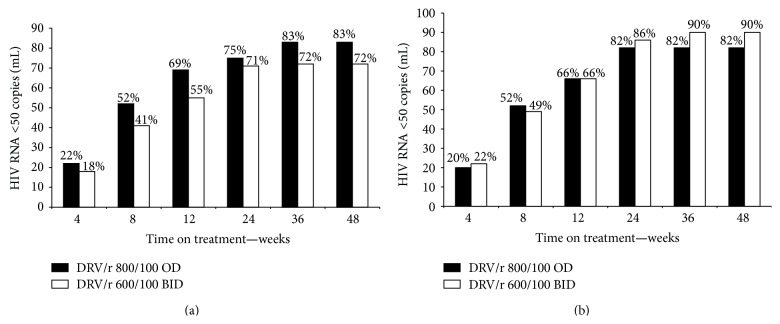
(a) HIV-1 RNA suppression on DRV/r-based second-line treatment, by dose. Patients with HIV-1 subtype B virus. (b) HIV-1 RNA suppression on DRV/r-based second-line treatment, by dose. Patients with HIV-1 subtype non-B virus.

By week 48, there were 111/135 (82%) patients in the DRV/r 800/100 mg OD arm with HIV-1 RNA suppression below 50 copies/mL (Intent to Treat TLOVR analysis) versus 109/137 (80%) patients in the DRV/r 600/100 mg BID arm (Table 2). The corresponding percentages of patients with HIV-1 RNA suppression below 400 copies/mL at week 48 were 118/137 (87%) in the DRV/r 800/100 mg OD arm and 115/137 (84%) in the DRV/r 600/100 BID arm. None of the patients developed treatment-emergent major protease resistance-associated mutations during the study.  Table 3 and Figure 1 show the percentage of patients with HIV-1 RNA <50 copies/mL over time by baseline HIV-1 subtype. There was no evidence for differences in efficacy of DRV/r at either dose between patients with subtype B and non-B infection. In addition, there was no clear correlation between HIV-1 RNA suppression at week 48 and either the number of active NRTIs used or baseline CD4 count (Table 4).

### 3.3. Safety Analysis

Table 5 shows the safety profile up to week 48 by treatment arm. There was a trend for fewer clinical adverse events in the DRV/r 800/100 mg OD arm compared with the 600/100 mg BID arm, including fewer serious adverse events (4% versus 7%), adverse events leading to permanent discontinuation of treatment (1% versus 4%), and Grades 2–4 adverse events (43% versus 48%). The most common Grades 1–4 adverse events were gastrointestinal side effects (diarrhoea, nausea, and vomiting), which tended to occur less frequently in the OD arm. There was no difference between the arms in the risk of Grade 3 or Grade 4 elevations in lipids or glucose.

## 4. Discussion

This exploratory analysis aimed to provide evidence of the virological efficacy of DRV/r in combination with at least two optimised NRTIs as second-line ART following failure of first-line NNRTI-based therapy. After 48 weeks of treatment, 82% of subjects showed HIV-1 RNA suppression below 50 copies/mL. The 800/100 mg once daily dose showed similar efficacy to the 600/100 mg twice daily dose, but with trends for fewer serious adverse events (4% versus 7%) and fewer discontinuations for adverse events (1% versus 4%). These findings support the use of DRV/r 800/100 mg once daily, in PI-naïve patients who have failed NNRTI-based therapy.

There are several limitations to this analysis, if the results are to be extrapolated to people failing NNRTI-based ART in low- or middle-income countries. First, the trial selection criteria could have excluded important groups of people who may need treatment with DRV/r, such as pregnant or breastfeeding women or those with active AIDS defining illnesses or laboratory abnormalities. Clinical trials of DRV/r in such settings should have as few selection criteria as possible in order to mirror real-life clinical practice. Secondly, the patients were tested for drug resistance at baseline, and their background NRTI treatment was optimized based on the results. Routine testing for drug resistance at the time of virological failure of first-line ART is not the current standard of care in most low-income countries, and this may have overestimated the efficacy of DRV/r in a setting where resistance testing is not affordable. Thirdly, there was no comparison with other PI/r-based regimens widely used in low- or middle-income countries and it is therefore not possible to estimate potential differences in efficacy or safety between DRV/r and alternative treatments. Finally, this was a post hoc comparison of the treatment groups in the subset of PI-naïve patients; the trial was not powered to compare the treatments in this subgroup. Finally, there was a wide range of ages included in the study (18–77 years). The effects of darunavir/ritonavir in elderly patients who may have other comorbidities need to be studied in more detail.

The current WHO 2013 guidelines for resource-limited settings recommend second-line treatment with a PI/r combined with two NRTIs [4]. Use of NRTIs to accompany the DRV/r regimens in ODIN (primarily tenofovir, zidovudine, and lamivudine) was consistent with current drug availability in these settings. Two large clinical trials (EARNEST and SECOND-LINE) have recently evaluated an alternative option, combining a PI with an integrase inhibitor to provide a second-line option that is not affected by preexisting NRTI resistance [14, 15]. These studies have shown similar efficacy when LPV/r was combined with either the integrase inhibitor raltegravir or NRTIs. This observation indicates that potential NRTI cross-resistance does not pose a significant concern, at least in the context of a clinical trial with relative short length of follow-up.

In conclusion, the PI-naïve patients in the ODIN study had similar rates of HIV-1 RNA suppression on the 800/100 mg once daily and 600/100 mg twice daily doses. The efficacy was also similar for patients with different HIV-1 subtypes and for those with 0, 1, or 2 active NRTIs in their treatment backbone. More randomised studies are needed to compare the efficacy of DRV/r, ATV/r, and LPV/r in second-line treatment of PI-naïve patients, in combination with either nucleoside analogues or integrase inhibitors.

## Figures and Tables

**Table 1:** Baseline characteristics of protease inhibitor naïve patients in the ODIN trial.

	Darunavir/ritonavir 800/100 mg once daily	Darunavir/ritonavir 600/100 mg twice daily
*n*	135	137
Age (years): median (range)	38 (18–70)	38 (18–77)
Female	49 (36.3%)	47 (34.3%)
CD4 count <200 cells/*µ*L	59 (43.7%)	54 (39.4%)
HIV-1 RNA ≥100,000 copies/mL	9 (6.7%)	13 (9.5%)
Race		
Black	35 (25.9%)	38 (27.3%)
Caucasian	27 (20.0%)	39 (28.5%)
Oriental/Asian	41 (30.4%)	29 (21.2%)
Other	32 (23.7%)	31 (22.6%)
Region		
Africa	22 (16.3%)	23 (16.8%)
Asia	41 (30.4%)	28 (20.4%)
Europe/Australia	11 (8.1%)	12 (8.8%)
N America	11 (8.1%)	16 (11.7%)
S America	50 (37.0%)	58 (42.3%)
CDC Stage 3 or 4	53 (39.3%)	50 (36.5%)
HIV-1 subtype		
B	64 (47.4%)	78 (56.9%)
A1	1 (0.7%)	0
C	26 (19.3%)	26 (19.0%)
CRF01_AE	37 (27.4%)	27 (19.7%)
CRF02_AG	2 (1.5%)	0
CRF012_BF	3 (2.2%)	2 (1.5%)
F1	2 (1.5%)	4 (2.9%)
Fully active NRTIs used^*∗*^		
0	7 (6%)	9 (7%)
1	34 (27%)	35 (27%)
≥2	85 (67%)	88 (67%)

All results shown are *n* (%), unless otherwise stated.

^*∗*^Baseline genotyping results were available. There were 9 patients in the 800/100 mg OD arm and 5 in the 600/100 mg BID arm who did not have genotypes available at the baseline visit.

**Table 2:** Efficacy results at week 48 for protease inhibitor naïve patients in the ODIN trial.

	Darunavir/ritonavir 800/100 mg once daily	Darunavir/ritonavir 600/100 mg twice daily
*n*	135	137
HIV-1 RNA <50 copies/mL	111 (82%)	109 (80%)
HIV-1 RNA <400 copies/mL	118 (87%)	115 (84%)

All results shown are *n* (%), unless otherwise stated.

**Table 3:** HIV-1 RNA suppression <50 copies/mL over time, by HIV-1 subtype.

	Darunavir/ritonavir 800/100 mg once daily	Darunavir/ritonavir 600/100 mg twice daily
HIV-1 subtype B	*n* = 64	*n* = 78

Week 4	14/64 (22%)	14/78 (18%)
Week 8	33/64 (52%)	32/78 (41%)
Week 12	44/64 (69%)	43/78 (55%)
Week 24	48/64 (75%)	55/78 (71%)
Week 36	53/64 (83%)	56/78 (72%)
Week 48	53/64 (83%)	56/78 (72%)

HIV-1 subtype non-B	*n* = 71	*n* = 59

Week 4	14/71 (20%)	13/59 (22%)
Week 8	37/71 (52%)	29/59 (49%)
Week 12	47/71 (66%)	39/59 (66%)
Week 24	58/71 (82%)	51/59 (86%)
Week 36	58/71 (82%)	53/59 (90%)
Week 48	58/71 (82%)	53/59 (90%)

All results shown are *n* (%), unless otherwise stated.

**Table 4:** HIV-1 RNA suppression at week 48, by number of active NRTIs and baseline CD4 count.

	Darunavir/ritonavir 800/100 mg once daily	Darunavir/ritonavir 600/100 mg twice daily
	*n* = 137	*n* = 135
HIV-1 RNA <50 copies/mL		
Number of active NRTIs		
0	6/7 (86%)	8/9 (89%)
1	30/34 (88%)	32/35 (91%)
≥2	64/85 (75%)	69/88 (78%)

HIV-1 RNA <400 copies/mL		
Number of active NRTIs		
0	6/7 (86%)	9/9 (100%)
1	31/34 (91%)	32/35 (91%)
≥2	64/85 (75%)	74/88 (84%)

HIV-1 RNA <50 copies/mL		
Baseline CD4 count		
<200 cells/*µ*L	49/59 (83%)	41/54 (76%)
≥200 cells/*µ*L	62/76 (82%)	68/83 (82%)

HIV-1 RNA <400 copies/mL		
Baseline CD4 count		
<200 cells/*µ*L	51/59 (86%)	44/54 (82%)
≥200 cells/*µ*L	67/76 (88%)	71/83 (86%)

**Table 5:** Safety results for protease inhibitor naïve patients in the ODIN trial.

Adverse event	Darunavir/ritonavir 800/100 mg once daily	Darunavir/ritonavir 600/100 mg twice daily
	*n* = 135	*n* = 137
Clinical adverse events		
Any serious adverse event	5 (4%)	9 (7%)
Any adverse event leading to permanent drug discontinuation	1 (1%)	6 (4%)
Any Grades 2–4 adverse events	58 (43%)	66 (48%)

Most common Grades 1–4 adverse events		
Diarrhoea	18 (13%)	34 (25%)
Nausea	14 (10%)	17 (12%)
Headache	11 (8%)	11 (8%)
Nasopharyngitis	6 (4%)	12 (9%)
Upper respiratory tract infection	6 (4%)	11 (8%)
Vomiting	3 (2%)	14 (10%)
Rash	9 (7%)	6 (4%)
Anorexia	2 (1%)	7 (5%)

Grades 3-4 lipid/glucose abnormalities		
Total cholesterol	4 (3%)	4 (3%)
LDL cholesterol	5 (4%)	4 (3%)
Triglycerides	4 (3%)	3 (2%)
Hyperglycemia	2 (1%)	3 (2%)

All results shown are *n* (%), unless otherwise stated.
